# Suprasellar pilocytic astrocytoma with leptomeningeal dissemination

**DOI:** 10.4102/sajr.v28i1.2876

**Published:** 2024-05-27

**Authors:** Siti S. Mohamad Zaini, Hilwati Hashim, Emy S. Rosani, Nor S. Bakar, Suryati Mohd Yusoff

**Affiliations:** 1 Department of Radiology, Hospital Kuala Lumpur, Kuala Lumpur, Malaysia; 2 Department of Radiology, Faculty of Medicine, Universiti Teknologi MARA, Sungai Buloh, Malaysia; 3 Department of Radiology, Hospital Sungai Buloh, Sungai Buloh, Malaysia; 4 Department of Pathology, Faculty of Medicine, Universiti Teknologi MARA, Sungai Buloh, Malaysia; 5 Department of Pathology, Hospital Kuala Lumpur, Kuala Lumpur, Malaysia

**Keywords:** pilocytic astrocytoma, leptomeningeal, suprasellar, low-grade tumour, metastasis

## Abstract

Leptomeningeal dissemination is a rare manifestation of pilocytic astrocytoma. It may occur with higher-grade tumours like medulloblastoma, ependymoma and high-grade glioma, but is extremely rare with low-grade glioma. There has been a growing number of reported cases documenting leptomeningeal dissemination of pilocytic astrocytoma in the medical literature.

**Contribution:**

Description of a World Health Organization (WHO) Grade I suprasellar pilocytic astrocytoma with leptomeningeal dissemination in the brain and spinal cord which showed progression of the leptomeningeal nodules without tumour upgrading on long-term follow-up.

## Introduction

Pilocytic astrocytomas (PA) are low-grade tumours, classified as circumscribed astrocytic glioma, World Health Organization (WHO) central nervous system (CNS) Grade I tumours according to the 2021 classification.^1^ They account for 15% of all paediatric brain tumours, the most common CNS tumour in children.^2^ They typically occur in children and young adults and have a good prognosis.^3^ With complete surgical resection, the 5-year survival rate is 94.1%.^2^ Leptomeningeal dissemination is a rare manifestation of pilocytic astrocytoma. This report describes a case of pilocytic astrocytoma with leptomeningeal brain and spinal involvement which demonstrated progression on follow-up.

## Case report

**FIGURE 1:**
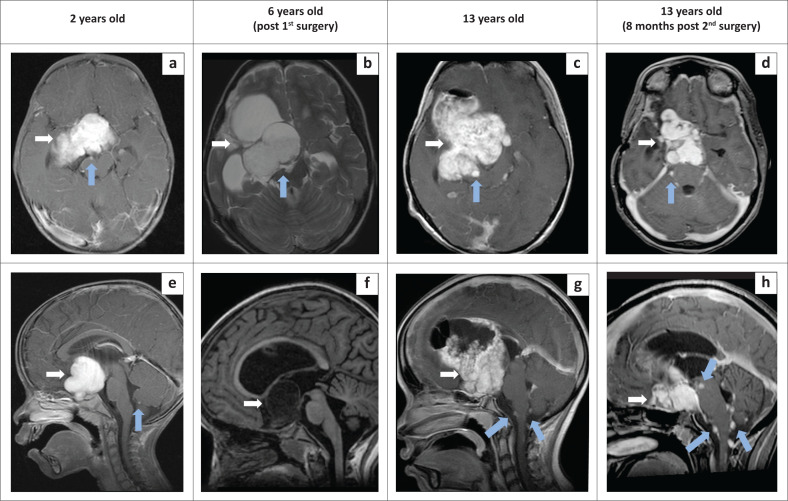
Series of axial (a, b, c, d upper row) and sagittal (e, f, g, h lower row) MR imaging over 11 years at age 2, age 6 and age 13. White arrow: suprasellar tumour. Blue arrow: leptomeningeal nodule. (a, e) Contrasted MRI brain at age 2 shows a large, solid, enhancing suprasellar mass with small enhancing leptomeningeal nodules at the right interpeduncular cistern (a) and anterior to the cerebellar vermis (e). (b, f) Repeat MRI brain at age 6 after partial tumour resection shows a smaller suprasellar mass causing obstruction of the right lateral ventricle. The leptomeningeal nodule at the right interpeduncular cistern is still present and appears isointense on T2WI (b). Contrast administration was declined at this time. (c, g) Repeat MRI brain at age 13 revealed that the residual suprasellar mass had increased in size and the leptomeningeal nodules increased in size and number in the right interpeduncular cistern (c) and anterior to the cerebellar vermis with new nodules at the craniocervical junction (d). (f, h) Repeat MRI at 8 months post surgery showed a decrease in the size of the suprasellar mass with an increase in the size and number of leptomeningeal nodules.

A 13-year-old female patient first presented at the age of 2 years with abnormal movement of her right eye following a fall. She had a background history of failure to thrive. Endocrinology evaluations revealed a diagnosis of partial hypopituitarism with cranial diabetes insipidus and hypocorticolism, for which she was prescribed oral Hydrocortisone and Levothyroxine. Unfortunately, the patient’s parents defaulted treatment and follow-up appointments. An MRI of the brain conducted at that time showed a large lobulated suprasellar mass with intense enhancement on post-contrast imaging (Figure 1a & 1e). The mass infiltrated the hypothalamus, right side of midbrain, right medial temporal lobe and right basal ganglia. It extended inferiorly to the sellar region, resulting in compression of the pituitary gland. There was mild mass effect on the third ventricle, leading to midline shift but no obstructive hydrocephalus. At this point, there were already several small enhancing leptomeningeal nodules detected at the right interpeduncular cistern and anterior to the cerebellar vermis.

**FIGURE 2:**
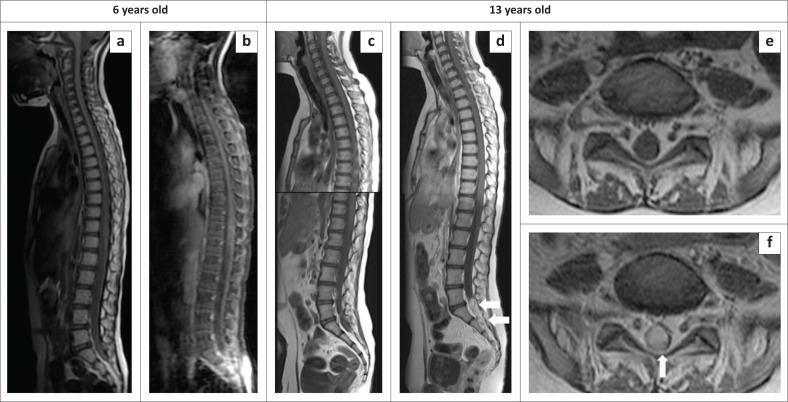
Series of MR imaging of the spine at ages 6 and 13 years. Spinal MRI at age 6. (a) Sagittal T1W pre-contrast and (b) T1W post-contrast fat saturated sequence. The imaging does not reveal any spinal lesions. Spinal MRI at age 13. (c) Sagittal and (e) Axial T1W pre-contrast. (d) Sagittal and (f) Axial T1W fat saturated post-contrast. There is an enhancing intraspinal dural lesion (white arrows) at the level of L5 extending to S2, suggestive of leptomeningeal dissemination.

As the patient defaulted treatment, no intervention was undertaken at the initial presentation. Four years later, at age 6, she presented at a different hospital with bilateral eye blindness and left-sided hemiparesis. A partial tumour resection was performed, and tissue biopsy revealed a suprasellar pilocytic astrocytoma WHO Grade I. Post operative MRI of the brain and spine revealed a smaller suprasellar mass with a stable leptomeningeal nodule at the right interpeduncular cistern (Figure 1b & 1f). No significant findings were observed in the spine (Figure 2a & 2b). The mass caused obstructive hydrocephalus, necessitating the insertion of a ventriculoperitoneal shunt. Subsequently, she underwent a complete cycle of chemotherapy, before defaulting treatment again. Fortunately, she did not develop any new symptoms and was able to attend normal school and read Braille.

She re-presented at 13 years of age with symptoms of increased intracranial pressure and lethargy for 2 weeks and a seizure on the day of presentation. Repeat MRI brain (Figure 1c & 1g) showed that the residual suprasellar mass had increased in size with a solid-cystic appearance and extension to the right frontotemporal lobe, right midbrain and prepontine region, causing mass effect. There was also an increase in the number and size of the leptomeningeal nodules at the right interpeduncular cistern and anterior to the cerebellar vermis with new nodules at the craniocervical junction. Cyst aspiration and tumour debulking was performed. Post operatively, the hydrocephalus and mass effect resolved, and the patient remained well. She was discharged on day 14 post-surgery, with residual left-sided hemiparesis and bilateral eye blindness.

**FIGURE 3:**
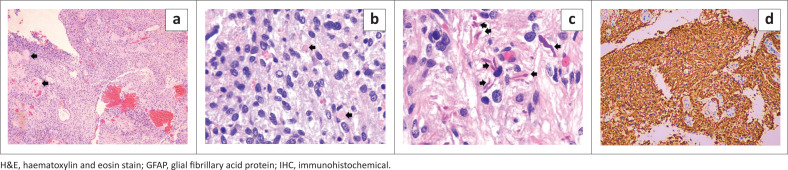
(a) Low magnification histopathological examination showing a moderately cellular tumour with hyaline vessels (black arrows) (H&E x40). (b) High magnification showing tumour cells with oval nuclei and eosinophilic globules (black arrows.) (H&E x400). (c) Higher magnification of the tumour indicating piloid astrocytic tumour cells with long delicate hair-like processes and eosinophilic Rosenthal fibres (black arrows) characteristic of pilocytic astrocytoma (H&E x600). (d) Immunohistochemical (IHC) stain showed brown staining of the tumour cells with GFAP positivity confirming glial tumour differentiation or origin (IHC x 40).

Microscopic examination of the surgical specimen showed a moderately cellular glial tumour with a biphasic pattern; compact piloid tumour areas interspersed with loose microcytic areas. Hyalinized blood vessels were noted (Figure 3a). Eosinophiloc globules (Figure 3b) and Rosenthal fibres (Figure 3c) were noted. The tumour was positive for glial fibrillary acid protein (Figure 3d) that confirmed astrocytic glial differentiation or origin. Mitotic activity was not increased; the Ki-67 index was low, less than 2%. The final diagnosis was pilocytic astrocytoma (WHO Grade I).

At 8 months post-surgery, a repeat MRI of the brain and spine (Figure 1d & 1h and Figure 2c–2f) revealed a reduction in the size of the suprasellar mass with less mass effect. However, the leptomeningeal nodules in the brain and craniocervical junctions had increased in size and number, without significant mass effect on the brainstem or narrowing of the foramen magnum. Additionally, a new enhancing dural lesion at the lumbosacral region suggested leptomeningeal metastases. No adjuvant treatment was initiated at this juncture, and unfortunately, the patient succumbed at home a few days following the MRI examination.

## Discussion

Pilocytic astrocytoma is a WHO grade 1 tumour. The diagnosis is confirmed by histopathological examination integrated with molecular genetic biomarkers of the biopsied tumour tissue.^1^ The tumour comprises piloid tumour cells exhibiting bland oval nuclei with wavy fibrillary processes. Piloid refers to “hair-like,” long, bipolar cytoplasmic processes. The presence of Rosenthal fibres and eosinophilic globules are characteristic. It is important to look for a pilomyxoid element in pilocytic astrocytoma as its presence is associated with a less favourable prognosis. Microscopically, pilomyxoid astrocytoma is characterized by a monotonous population of bipolar cells dispersed in a myxoid background with an absence of Rosenthal fibres and granular bodies. The presented case did not demonstrate a pilomyxoid element. There is no particular predilection of tumour cells for an angiocentric arrangement, i.e., no well-defined increase in cell density around vascular structures.^4^ Immunohistochemistry staining reveals positivity for glial fibrillary acid protein (GFAP). Strong and diffuse positivity to GFAP favours PA as pilomyxoid astrocytoma generally shows a variable staining pattern.^5^ Genetic findings may reveal BRAF alterations with lack of IDH mutations and TP53 mutations.^6^ However, this test was omitted in the current case due to the high cost.

As mentioned, the prognosis is mostly good.^7^ The tumour usually occurs near the midline, commonly arising in the cerebellum, at the optic nerve and chiasm, or in the hypothalamus-thalamus region. Less common locations include the cerebral hemispheres, the cerebral ventricles, velum interpositum, and spinal cord.^8,9,10,11^

Although it is classified as Grade I tumour, it may behave atypically either as local recurrence or, occasionally with malignant transformation and metastatic spread.^8,9,12^ Low-grade primary tumour of the CNS with cerebrospinal fluid (CSF) leptomeningeal spread is uncommon. It may occur with tumours like medulloblastomas (WHO Grade IV), ependymomas and high-grade gliomas, but is extremely rare in low-grade gliomas.^13^ A limited number of cases with dissemination of low-grade gliomas have been reported.^10,11^ Leptomeningeal dissemination of PA in children is even rarer,^8,10,12^ however, an increasing number of cases have been documented. In 2013, Bian et al. reported 6 cases of leptomeningeal dissemination of PA.^14^ They also peer-reviewed the published literature and found a total of 53 documented cases of disseminated PA with the most common being leptomeningeal dissemination, reported in over 88% of cases.

The most common presenting symptom of the hypothalamic-optic pathway PA is altered visual acuity as noted in the presented patient. Endocrine dysfunction, usually short stature related to decreased growth hormone, is also seen.^15^ In the current case, mass effect on the pituitary gland caused partial hypopituitarism. Large tumours are usually heterogeneous and mainly solid, with mild or marked enhancement. Hydrocephalus occurs in suprasellar tumours secondary to extension into the anterior portion of the third ventricle and obstruction at the foramen of Monro, which was seen in the presented case.^16^ Almost similar findings were also reported by Alyeldien et al.^7^ and Chang et al.^17^

Two other paediatric cases documented spinal dissemination in patients with optic chiasm astrocytomas.^13,15^ The possible mechanism could be due to contact of the tumour with the ventricular system which leads to tumour cell invasion of the perivascular and subarachnoid spaces, increasing the possibility of leptomeningeal dissemination.^18^ Leptomeningeal dissemination through a haematogenous route has also been suggested.^19^ Given the aggressive appearance of the tumour as in the presented case, a blood-brain barrier breach may have contributed to haematogenous leptomeningeal spread.

Considering that leptomeningeal metastases are reported in disseminated PA,^14^ it is important for patients to undergo routine spinal MRI as part of the imaging follow-up. This is especially important when the tumour displays aggressive characteristics or is in close proximity to CSF spaces, which can facilitate the dissemination. A multidisciplinary treatment strategy, inclusive of social support, is essential to mitigate patient non-adherence to treatment, as demonstrated in this case.

The mainstay of hypothalamic-optic pathway PA is surgical resection.^18^ In the current case, only partial tumour resection was performed as invasion into the hypothalamus precluded complete resection. Most patients with large tumours respond to platinum-based chemotherapy which may also improve serious visual disturbances. First-line management with chemotherapy can preserve cognitive outcomes and may avoid the need for radiotherapy.^18^ Combined chemo- and radiation therapy are effective at disease control, with an overall 5-year survival of > 90%.^20^

## Teaching point

Leptomeningeal spread is a rare manifestation of PA even though it is classified as Grade I tumour. For early detection of multifocal spread of the tumour, long-term follow-up is important. MRI of the spine should be performed when the primary PA is located in proximity to CSF spaces as it increases the possibility of leptomeningeal dissemination.
